# Features and outcomes of bailout repeat transcatheter aortic valve implantation (TAVI): the Bailout Acute TAVI-in-TAVI to Lessen Events (BATTLE) international registry

**DOI:** 10.1007/s00392-023-02239-8

**Published:** 2023-06-09

**Authors:** Arturo Giordano, Nicola Corcione, Marco Barbanti, Giuliano Costa, Elena Dipietro, Ignacio J. Amat-Santos, Javier Gómez-Herrero, Azeem Latib, Andrea Scotti, Luca Testa, Francesco Bedogni, Andreas Schaefer, Marco Russo, Francesco Musumeci, Paolo Ferraro, Alberto Morello, Michele Cimmino, Michele Albanese, Martino Pepe, Salvatore Giordano, Giuseppe Biondi-Zoccai

**Affiliations:** 1 Unità Operativa di Interventistica Cardiovascolare, Pineta Grande Hospital, Castel Volturno, Italy; 2 Unità Operativa di Emodinamica, Santa Lucia Hospital, San Giuseppe Vesuviano, Italy; 3 Division of Cardiology, Università degli Studi di Enna “Kore”, Enna, Italy; 4 Division of Cardiology, A.O.U. Policlinico “Rodolico-San Marco”, University of Catania, Catania, Italy; 5 Department of Cardiology, CIBERCV, University Clinical Hospital of Valladolid, Valladolid, Spain; 6 Department of Cardiology, Montefiore Medical Center, New York, NY USA; 7 Cardiology Unit, IRCCS Policlinico San Donato, Milan, Italy; 8 Department of Cardiovascular Surgery, University Heart and Vascular Center of Hamburg, Hamburg, Germany; 9 Department of Cardiac Surgery and Heart Transplantation, San Camillo Forlanini Hospital, Rome, Italy; 10 Cardiovascular Diseases Section, Department of Interdisciplinary Medicine (DIM), University of Bari Aldo Moro, Bari, Italy; 11 Division of Cardiology, Department of Medical and Surgical Sciences, Magna Graecia University, Catanzaro, Italy; 12 Department of Medico-Surgical Sciences and Biotechnologies, Sapienza University of Rome, Latina, Italy; 13 Mediterranea Cardiocentro, Naples, Italy

**Keywords:** Aortic stenosis, Bailout, TAVI-in-TAVI, TAVR-in-TAVR, Transcatheter aortic valve implantation, Transcatheter aortic valve replacement

## Abstract

**Aim:**

Transcatheter aortic valve implantation (TAVI) is a mainstay in the management of severe aortic stenosis in patients with intermediate to prohibitive surgical risk. When a single TAVI device fails and cannot be retrieved, TAVI-in-TAVI must be performed acutely, but outcomes of bailout TAVI-in-TAVI have been incompletely appraised. We aimed at analyzing patient, procedural and outcome features of patients undergoing bailout TAVI-in-TAVI in a multicenter registry.

**Methods:**

Details of patients undergoing bailout TAVI-in-TAVI, performed acutely or within 24 h of index TAVI, in 6 international high-volume institutions, were collected. For every case provided, 2 same-week consecutive controls (prior TAVI, and subsequent TAVI) were provided. Outcomes of interest were procedural and long-term events, including death, myocardial infarction, stroke, access site complication, major bleeding, and reintervention, and their composite (i.e. major adverse events [MAE]).

**Results:**

A total of 106 patients undergoing bailout TAVI-in-TAVI were included, as well as 212 controls, for a total of 318 individuals. Bailout TAVI-in-TAVI was less common in younger patients, those with higher body mass index, or treated with Portico/Navitor or Sapien devices (all p < 0.05). Bailout TAVI-in-TAVI was associated with higher in-hospital rates of death, emergency surgery, MAE, and permanent pacemaker implantation (all p < 0.05). Long-term follow-up showed that bailout TAVI-in-TAVI was associated with higher rates of death and MAE (both < 0.05). Similar findings were obtained at adjusted analyses (all p < 0.05). However, censoring early events, outlook was not significantly different when comparing the two groups (p = 0.897 for death, and p = 0.645 for MAE).

**Conclusions:**

Bail-out TAVI-in-TAVI is associated with significant early and long-term mortality and morbidity. Thus, meticulous preprocedural planning and sophisticated intraprocedural techniques are of paramount importance to avoid these emergency procedures.

**Supplementary Information:**

The online version contains supplementary material available at 10.1007/s00392-023-02239-8.

## Introduction

Transcatheter aortic valve implantation (TAVI), also known as transcatheter aortic valve replacement (TAVR), is a risk-beneficial and cost-beneficial intervention in patients with severe aortic valve disease at intermediate to prohibitive risk and therefore a reasonable alternative to surgical aortic valve replacement (SAVR) [1, 2].

While TAVI is often technically successful and uneventful, a transcatheter heart valve (THV) occasionally fails to achieve adequate results, especially in terms of significant aortic regurgitation. In such cases, bailout TAVI-in-TAVI is an adequate treatment option [3]. Indeed, TAVI-in-TAVI represents a clinical dilemma, whenever aortic regurgitation is significant, but not exceedingly severe [4].

To date, several reports focus on elective TAVI-in-TAVI, i.e. procedures performed not because of acute failure of a TAVI device, during the index procedure, but weeks, months or years afterwards [4–6], and extensive clinical evidence is available on TAVI after SAVR [7–9]. Conversely, studies focusing on bailout TAVI-in-TAVI are typically of small size, limited follow-up, with some notable exceptions [3, 10]. Indeed, Makkar et al. provided insightful results on 63 patients requiring bailout TAVI-in-TAVI when using early generation balloon-expandable devices, highlighting patient features and mid-term prognosis [11]. Nonetheless, the evidence base of bailout TAVI-in-TAVI in the current era of new-generation THV is scant.

We conducted a retrospective observational controlled study aimed at appraising patient, procedural, and outcome features of patients undergoing bailout TAVI-in-TAVI.

## Methods

Several high-volume international TAVI centers were invited to participate to the Bailout Acute TAVI-in-TAVI to Lessen Events (BATTLE) international registry, providing retrospective patient, procedural, and outcome data. After formal acceptance of participation, a common protocol was designed and agreed upon, together with a anonymized dataset. The only inclusion criteria was bail-out TAVI-in-TAVI performed during the same procedure of index TAVI for native aortic stenosis, or within 24 h after procedure completion, and eligibility for at least 12-month follow-up.

Notably, for every case provided, 2 same-week consecutive controls (prior case, and subsequent case) were provided as well, again limiting inclusion to TAVI for native aortic stenosis.

Individual centers were responsible for obtaining consent and institutional review board for data collection and analysis, whereas data provided by Pineta Grande Hospital were extracted from the RISPEVA registry (https://clinicaltrials.gov/ct2/show/NCT02713932), already approved by the competent ethics committee of Pineta Grande Hospital, Castel Volturno, Italy.

Patient selection and procedural strategy, including device choice and indications for bailout TAVI-in-TAVI, were at operator’s discretion, as were ancillary medical management and follow-up procedures. Several clinically relevant endpoints were appraised, applying Valve Academic Research Consortium (VARC)-2 definitions whenever appropriate, focusing in particular on the following outcomes: device success, procedural success, death, myocardial infarction, stroke, access site complication, bleeding, major bleeding, emergency surgery, pacemaker implantation, tamponade, repeat TAVI during follow-up, repeat SAVR during follow-up, and major adverse event (the composite of death, myocardial infarction, stroke, major bleeding, or valve reintervention).

Descriptive analysis was based on reporting mean ± standard deviation for continuous variables, and count (%) for categorical variables. Bivariate analysis was based on unpaired Student t test for continuous variables and Fisher exact test for categorical variables. Survival analysis was based on the Kaplan–Meier method and log-rank test. In addition, Cox proportional hazard analysis was performed, computing hazard ratios, with corresponding 95% confidence intervals. Adjusted analysis was based on inverse probability of treatment weighting, applying weights to binary logistic regression, computing odds ratios, with corresponding 95% confidence intervals, and Cox proportional hazard analysis models. Statistical significance was set at the 2-tailed 0.05 level, without multiplicity adjustment. Computations were performed with Stata 13 (StataCorp, College Station, TX, USA).

## Results

A total of 106 patients requiring bailout TAVI-in-TAVI were included, as well as 212 controls. This amounted to a 1.6% rate (95% confidence interval 1.0–2.4%) when considering the total number of TAVI for native aortic stenosis during the study period. Patients requiring bailout TAVI-in-TAVI were significantly older (80.3 ± 6.5 in controls vs 82.2 ± 6.4 years in the TAVI-in-TAVI group, p = 0.012), and presented a smaller body mass index (27.5 ± 4.5 vs 26.4 ± 3.9 kg/m^2^), p = 0.037), but all other baseline patient features were similar in the 2 groups (Table 1). Focusing on imaging and other features, significant calcification was also significantly more common in patients requiring bailout TAVI-in-TAVI (111 [52.4%] vs 76 [71.7%], p = 0.001), who also exhibited more frequently low flow-low gradient aortic stenosis (10 [9.4%] vs 7 [3.3%], p = 0.032) and moderate or severe aortic regurgitation (111 [52.4%] vs 76 [71.7%], p = 0.001), had larger sinuses of Valsalva (31 ± 4 vs 33 ± 4 mm, p = 0.014), sino-tubular junction (28 ± 3 vs 30 ± 5 mm, p = 0.008), and ascending aortic diameters (34 ± 5 vs 36 ± 5 mm, p = 0.014 (Table 1S).

**Table 1:** Baseline features

Features	Control	TAVI-in-TAVI	P value
Patients	212	106	–
Age (years)	80.3 ± 6.5	82.2 ± 6.4	0.012
Female	129 (60.9%)	55 (51.9%)	0.148
Body mass index (kg/m^2^)	27.5 ± 4.5	26.4 ± 3.9	0.037
Diagnosis			0.151
Aortic stenosis	186 (87.7%)	91 (85.6%)	
Aortic regurgitation	1 (0.5%)	3 (2.8%)	
Mixed aortic valve disease	24 (11.3%)	10 (9.4%)	
Failed bioprosthesis	1 (0.5%)	2 (1.9%)	
Risk			0.534
Inoperable	2 (0.9%)	2 (1.9%)	
High	24 (11.3%)	16 (15.1%)	
Intermediate	30 (14.2%)	17 (16.0%)	
Low	156 (73.6%)	71 (67.0%)	
Logistic EuroSCORE	14.8 ± 12.5	14.9 ± 11.6	0.943
EuroSCORE_II	4.7 ± 4.9	4.9 ± 5.1	0.710
Coronary artery disease	74 (34.9%)	43 (40.6%)	0.327
Prior percutaneous coronary intervention	51 (24.1%)	23 (21.7%)	0.675
Prior coronary artery bypass grafting	18 (8.5%)	9 (8.5%)	1
Peripheral artery disease	24 (11.3%)	15 (14.2%)	0.473
Chronic obstructive pulmonary disease	42 (19.8%)	24 (22.6%)	0.560
New York Heart Association class			0.160
I	9 (4.3%)	4 (3.9%)	
II	75 (35.9%)	27 (26.2%)	
III	118 (56.5%)	64 (62.1%)	
IV	7 (3.4%)	8 (7.8%)	
Baseline eGFR	52 ± 21	52 ± 24	0.988

*TAVI* transcatheter aortic valve implantation

Procedural features differed significantly for device type, with bailout TAVI-in-TAVI being less frequent in patients treated with Portico/Navitor or Sapien (respectively 34 [16.0%] in controls vs 9 [8.5%] in TAVI-in-TAVI groups, and 44 [20.8%] vs 9 [8.5%], overall p = 0.004), and thus also those receiving a balloon-expandable valve as first device (46 [21.7%] vs 11 [10.4%], p = 0.013) (Table 2). Among reported causes of TAVI-in-TAVI, cranial migration and significant aortic regurgitation were the most common (Table 2S), whereas a device of the same type/brand was used in most cases (Table 3S), with upsizing being required in 17 (16.0%) of procedures. Large aortic valve dimensions or significant aortic regurgitation at baseline were slightly more common in the TAVI-in-TAVI group (24 [22.6%] vs 39 [18.4%], p = 0.374), and subjects with these features requiring TAVI-in-TAVI more commonly required non-femoral access (including aortic, apical and axillary, p = 0.006).

**Table 2:** Procedural features

Features	Control	TAVI-in-TAVI	P value
Patients	212	106	–
Approach			0.082
Aortic or apical	0	3 (2.8%)	
Axillary	5 (2.4%)	3 (2.8%)	
Femoral	207 (97.6%)	100 (94.3%)	
General anesthesia	9 (4.3%)	7 (6.6%)	0.417
Embolic protection device	4 (1.9%)	3 (2.8%)	0.690
Predilation	166 (82.2%)	82 (79.6%)	0.642
First device			0.004
Accurate	24 (11.3%)	13 (12.3%)	
Allegra	3 (1.4%)	5 (4.7%)	
CoreValve	54 (25.5%)	37 (34.9%)	
Evolut	51 (24.1%)	29 (27.4%)	
Jena Valve	0	2 (1.9%)	
Myval	2 (0.9%)	2 (1.9%)	
Portico/Navitor	34 (16.0%)	9 (8.5%)	
Sapien	44 (20.8%)	9 (8.5%)	
First device balloon-expandable	46 (21.7%)	11 (10.4%)	0.013
First device size (mm)	26.6 ± 2.6	27.2 ± 2.8	0.053
Second device different from first one	0	19 (20.2%)	–
Upsizing of second device	0	17 (16.0%)	–
Second device size (mm)	–	27.5 ± 2.9	–
Postdilation	54 (25.5%)	43 (40.6%)	0.007
Surgical hemostasis	6 (2.8%)	5 (4.7%)	0.516
Contrast volume (mL)	182 ± 102	249 ± 131	< 0.001
Fluoroscopy time (minutes)	24 ± 13	35 ± 14	< 0.001
Procedural time (hours)	1.5 ± 1.1	2.5 ± 2.4	< 0.001
Procedural success	211 (99.5%)	91 (85.9%)	< 0.001
Pacemaker dependency	25 (11.8%)	23 (21.7%)	0.030

*TAVI* transcatheter aortic valve implantation

Expectedly, postdilation was performed more frequently in the bailout TAVI-in-TAVI group (54 [25.5%] vs 43 [40.6%], p = 0.007, with similarly significant differences for contrast volume (182 ± 102 vs 249 ± 131 mL, p < 0.001), fluoroscopy time (24 ± 13 vs 35 ± 14 min, p < 0.001), procedural time (1.5 ± 1.1 vs 2.5 ± 2.4 h, p < 0.001), procedural success (211 [99.5%] vs 91 [85.9%], p < 0.001), and pacemaker dependency (25 [11.8%] vs 23 [21.7%], p = 0.030).

**Fig. 1:**
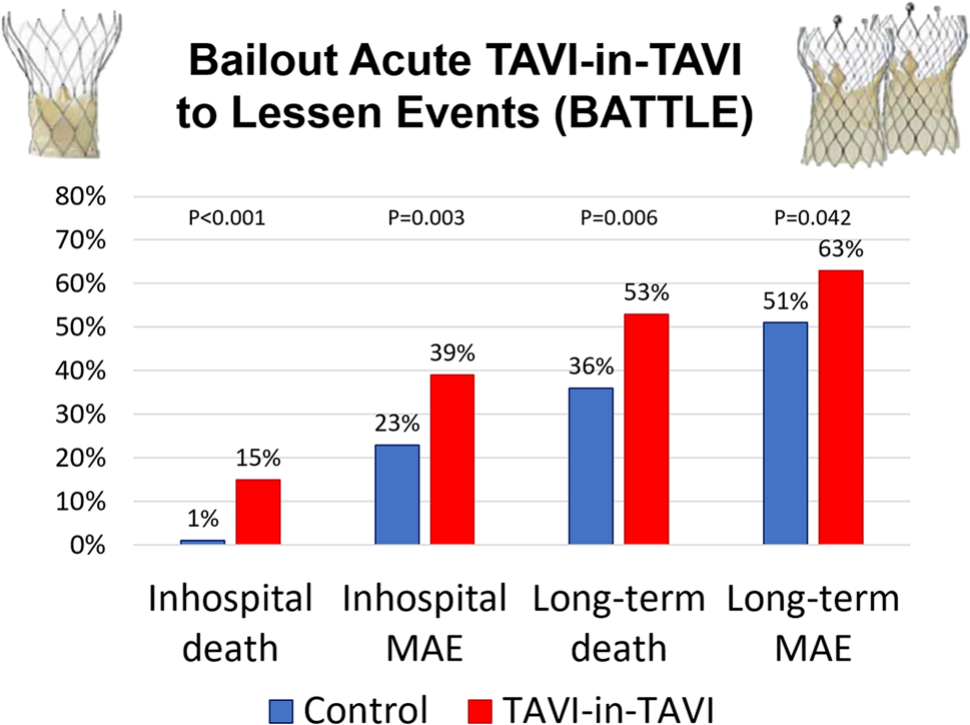
Summary of inhospital and long-term event rates. *MAE* major adverse event, *TAVI* transcatheter aortic valve implantation

Early outcomes were significantly worse in the bailout TAVI-in-TAVI group, including death (3 [1.4%] vs 16 [15.1%], p < 0.001), emergency surgery (1 [0.5%] vs 4 [3.8%], p = 0.044), pacemaker implantation (20 [9.4%] vs 23 [21.7%], p = 0.005), and major adverse event (48 [22.6%] vs 41 [38.7%], p = 0.003) (Table 4S; Fig. 1). The most common causes of inhospital death in patients undergoing TAVI-in-TAVI (N = 16) were irreversible heart failure (9 [56.3%]), cardiac arrest during the procedure (2 [12.5%]), and septic shock (2 [12.5%]). Other causes of fatalities were coronary occlusion, stroke, and tamponade (each occurring in 1 patient each [6.3%]).

**Fig. 2:**
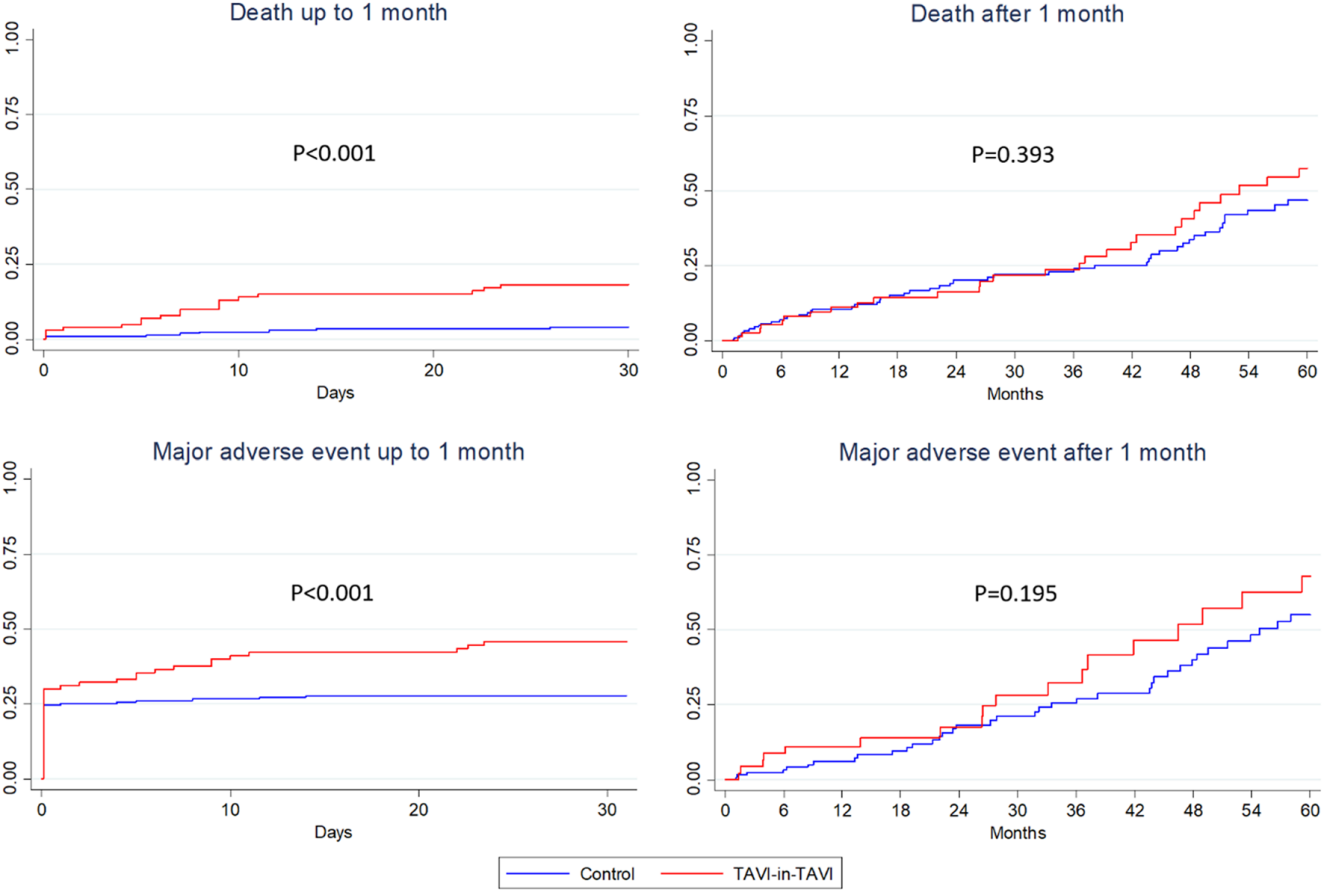
Failure curves for death and major adverse event, distinguishing events occurring within and after 1 month. *TAVI* transcatheter aortic valve implantation

Long-term follow-up (mean 2.3 ± 2.5 years, available at 1 year in 190 [59.6%], at 2 years in 138 [43.4%], and at 3 years in 108 [34.0%]) confirmed the significant increase for risk of death (77 [36.3%] vs 56 [52.8%], p = 0.006), as well as for major adverse event (108 [50.9%] vs 67 [63.2%], p = 0.042) (Table 3; Figs. 2; 1S). Notably, in no case significant coronary obstruction was reported after discharge in either group (whereas during the index hospitalization it had occurred in 3 cases, all in the control group). Follow-up assessments suggested similar valve performance over time, without differences in gradients or degree of regurgitation (Table 5S). Notably, no significant differences after censoring early outcomes were apparent in the 2 groups (Table 6S).

**Table 3:** Long-term outcomes

Features	Control	TAVI-in-TAVI	P value
Patients	212	106	–
Follow-up (years)	2.4 ± 2.4	2.2 ± 2.5	0.554
Death	77 (36.3%)	56 (52.8%)	0.006
Cardiovascular death	48 (22.6%)	31 (29.3%)	0.217
Myocardial infarction	8 (3.8%)	5 (4.7%)	0.766
Stroke	18 (8.5%)	8 (7.6%)	0.832
Bleeding	43 (20.3%)	28 (26.4%)	0.253
Major bleeding	23 (10.9%)	15 (14.2%)	0.463
Aortic valve reintervention	1 (0.5%)	0	1
Significant coronary obstruction	3 (1.4%)	0	0.553
Major adverse event^a^	108 (50.9%)	67 (63.2%)	0.042

*TAVI* transcatheter aortic valve implantation

^a^Composite of death, myocardial infarction, stroke, major bleeding, or aortic valve reintervention

Multivariable adjusted analysis confirmed the significant detrimental impact of bailout TAVI-in-TAVI on the risk of in-hospital death (odds ratio = 16.24 [95% confidence interval: 2.01–131.44], p = 0.009) and major adverse event (odds ratio = 2.34 [95% confidence interval: 1.26–4.36], p = 0.007), as well as long-term death (hazard ratio = 1.93 [95% confidence interval: 1.19–3.12], p = 0.007), and major adverse events (hazard ratio = 1.80 [95% confidence interval: 1.19–2.72], p = 0.005) (Table 7S).

Similar effect estimates were obtained in models based on inverse probability of treatment weighting (all p < 0.05). Intriguingly, models based on censoring of early events were instead non-significant (p = 0.897 for death, and p = 0.645 for major adverse events).

## Discussion

Developments in transcatheter valve procedures have been momentous, and TAVI, despite being still in its young adulthood, appears mature in its established benefits on morbidity and mortality [1]. A key technical dilemma has been since inception the optimal positioning of the TAVI device, as lower landing is typically more stable but may lead to higher pacemaker implantation rates, whereas a higher seating may occasionally lead to device instability with eventual pop-up requiring bailout TAVI-in-TAVI or, in the worst case scenario, emergency surgery [8, 9, 12].

We aimed at exploring the early and long-term outlook of patients requiring acute, unplanned, bailout TAVI-in-TAVI implantation. A multicenter collaboration was instituted, and contemporary control cases also collected to inform on potentially relevant predisposing factors among baseline and procedural features. Most importantly, a pragmatic case–control design enabled us to appraise early and long-term outcomes. We eventually compiled the largest dedicated series ever on this topic. We found that bailout TAVI-in-TAVI was relatively uncommon (1–2% of all TAVI cases), and was slightly more frequent in patients who were older, leaner, and with more calcific valves larger aortic root valve dimensions. Most importantly, bailout TAVI-in-TAVI was relatively more frequent when using self-expandable devices, especially some specific ones, but could still occur with any device.

The impact of bailout TAVI-in-TAVI was immediately dire, with an increased risk of several adverse inhospital outcomes, including death, emergency surgery, major adverse event, and pacemaker implantation. Such prognostically relevant differences were carried out up to long-term follow-up, such as bailout TAVI-in-TAVI conferred a significantly increased risk of death even several years after the index procedure, and the same applied to major adverse event. Yet, after the inhospital phase, differences in outcomes remained stable, without further separation of failure curves. Conversely, valve performance appeared satisfactory in both groups during follow-up.

In the quest for the optimization of TAVI outcomes, an apparently banal issue such as device positioning holds a key role in maximizing long-term benefits of TAVI, while minimizing complications. Evidently, an overly lower deployment of the device may lead to a moderate increase in the need for permanent pacemaker implantation, and possibly residual regurgitation. Yet, an overly higher positioning may lead to device malpositioning, instability, or even dislocation, possibly requiring bailout TAVI-in-TAVI [13]. Of course, several other factors may come into play in causing bailout TAVI-in-TAVI, such as anatomic features, operator’s experience, predilation, device choice, and postdilation. Accordingly, the debate can currently be epitomized as lower equals more pacemakers, and upper equals more bailout TAVI-in-TAVI [14].

Our findings may inform decision-makers by confirming prior reports and smaller series, that operators wishing to minimize the risk of bailout TAVI-in-TAVI should pay particular attention to old patients, those with smaller body mass index, more calcific valves, larger valvular or aortic diameters, and when considering the use of self-expandable devices, in particular some of them [15]. Furthermore, In case bailout TAVI-in-TAVI is eventually needed, utmost care should be put for peri-procedural and early inpatient care, being aware that the adverse prognostic impact of bailout TAVI-in-TAVI while being quite evident, is likely mechanistically limited to the procedural and postprocedural time frames.

While this topic has already been covered in some detail, either in sub-analyses of larger studies or dedicated series, our series is the largest and most comprehensive ever on the issue of acute bailout TAVI-in-TAVI. Accordingly, it confirms prior similar works expanding their scope and informing management strategy and patient care. While individualized decision-making remains crucial to maximize the early and long-term benefits of TAVI, while simultaneously minimizing its risks, our recommendation is to accept occasionally an overly inferior position of the TAVI device, as overly precise positioning may be impossible, and trying to minimize septal seating may lead to prosthesis displacement, eventually leading to much feared complications, including fatal ones [16–18].

Limitations of this work include the observational case–control design, the retrospective score, and the inclusion of several different generations of different TAVI devices [19, 20]. Accordingly, implications are valid in general terms but may not hold true for some specific device types, which have limited representation. In addition, while the sample is the largest ever collected on the topic, it is still only moderate in size. However, assuming a 1–2% rate of bailout TAVI-in-TAVI, only analysis of a dataset spanning dozens of thousands of patients could provide meaningfully larger numbers of patients.

In conclusion, bail-out TAVI-in-TAVI is associated with significant early and long-term mortality and morbidity, and should be prevented and avoided whenever possible. Indeed, meticulous preprocedural planning and sophisticated intraprocedural techniques are of paramount importance to avoid these emergency procedures.

### Supplementary Information

Below is the link to the electronic supplementary material.

Supplementary file1 (DOCX 92 kb)

## Data Availability

The datasets used and/or analyzed during the current study available from the corresponding author on reasonable request.

## References

[1] Giordano A, Biondi-Zoccai G, Frati G (2019). Transcatheter aortic valve implantation: clinical, interventional, and surgical perspectives.

[2] Barbanti M, Buccheri S, Rodés-Cabau J, Gulino S, Généreux P, Pilato G, Dvir D, Picci A, Costa G, Tamburino C, Leon MB, Webb JG (2017). Transcatheter aortic valve replacement with new-generation devices: a systematic review and meta-analysis. Int J Cardiol.

[3] Vrachatis DA, Vavuranakis M, Tsoukala S, Giotaki S, Papaioannou TG, Siasos G, Deftereos G, Giannopoulos G, Raisakis K, Tousoulis D, Deftereos S, Vavuranakis M (2020). TAVI: Valve in valve. A new field for structuralists? Literature review. Hellenic J Cardiol.

[4] Gallo M, Fovino LN, Blitzer D, Doulamis IP, Guariento A, Salvador L, Tagliari AP, Ferrari E (2022). Transcatheter aortic valve replacement for structural degeneration of previously implanted transcatheter valves (TAVR-in-TAVR): a systematic review. Eur J Cardiothorac Surg.

[5] Abdel-Wahab M, Kitamura M, Krieghoff C, Lauten P, Komatsu I, Thiele H, Holzhey D, Dvir D (2020). BASILICA for a degenerated self-expanding transcatheter heart valve: structural considerations for supra-annular prosthetic leaflets. JACC Cardiovasc Interv.

[6] Nascimento H, Rodrigues RA, Sousa C, Silva JC, Macedo F, Maciel MJ (2020). TAVI in TAVI: new paradigm. Acta Cardiol.

[7] Bruno F, Elia E, D'Ascenzo F, Marengo G, Deharo P, Kaneko T, Cuisset T, Fauchier L, De Filippo O, Gallone G, Andreis A, Fortuni F, Salizzoni S, La Torre M, Rinaldi M, De Ferrari GM, Conrotto F (2022). Valve-in-valve transcatheter aortic valve replacement or re-surgical aortic valve replacement in degenerated bioprostheses: a systematic review and meta-analysis of short and midterm results. Catheter Cardiovasc Interv.

[8] Nuis RJ, Benitez LM, Nader CA, Perez S, de Marchena EJ, Dager AE (2013). Valve-in-valve-in-valve transcatheter aortic valve implantation to treat a degenerated surgical bioprosthesis in a subaortic position. Tex Heart Inst J.

[9] Nuis RJ, Van Mieghem NM (2022). TAVI-in-TAVI: a new paradigm in case preparation. Eur Heart J Case Rep.

[10] Witkowski A, Jastrzebski J, Dabrowski M, Chmielak Z (2014). Second transcatheter aortic valve implantation for treatment of suboptimal function of previously implanted prosthesis: review of the literature. J Interv Cardiol.

[11] Makkar RR, Jilaihawi H, Chakravarty T, Fontana GP, Kapadia S, Babaliaros V, Cheng W, Thourani VH, Bavaria J, Svensson L, Kodali S, Shiota T, Siegel R, Tuzcu EM, Xu K, Hahn RT, Herrmann HC, Reisman M, Whisenant B, Lim S, Beohar N, Mack M, Teirstein P, Rihal C, Douglas PS, Blackstone E, Pichard A, Webb JG, Leon MB (2013). Determinants and outcomes of acute transcatheter valve-in-valve therapy or embolization: a study of multiple valve implants in the U.S. PARTNER trial (Placement of AoRTic TraNscathetER Valve Trial Edwards SAPIEN Transcatheter Heart Valve). J Am Coll Cardiol.

[12] Attisano T, Bellino M, Vigorito F, Maione A, Ravera A, Pierri A, Baldi C, Galasso G, Vecchione C, Bonan R (2022). Bioprosthetic valve fracture after TAVR complicated by balloon rupture: bail-out TAVR in TAVR in SAVR. JACC Case Rep.

[13] Chiariello GA, Villa E, Messina A, Troise G (2018). Dislocation of a sutureless prosthesis after type I bicuspid aortic valve replacement. J Thorac Cardiovasc Surg.

[14] Casenghi M, Oliva OA, Squillace M, Bellamoli M, Poletti E, Popolo Rubbio A, Testa L, Bedogni F, De Marco F (2021). Bailout from sinus jailing: in-series TAVR-in-TAVR to avoid coronary flow obstruction. JACC Case Rep.

[15] Ito N, Zen K, Kuwabara K, Matoba S (2021). Recapture failure in transcatheter aortic valve replacement with CoreValve Evolut R. Catheter Cardiovasc Interv.

[16] Mangieri A, Laricchia A, Montalto C, Palena ML, Fisicaro A, Cereda A, Sticchi A, Latib A, Giannini F, Khokhar AA, Colombo A (2021). Patient selection, procedural planning and interventional guidance for transcatheter aortic valve intervention. Minerva Cardiol Angiol.

[17] Morello A, Corcione N, Ferraro P, Cimmino M, Pepe M, Cassese M, Frati G, Biondi-Zoccai G, Giordano A (2021). The best way to transcatheter aortic valve implantation: from standard to new approaches. Int J Cardiol.

[18] Pagnotta P, Ferrante G, Presbitero P (2014). Rescue, "valve in valve" implantation after late onset CoreValve cusp rupture leading to acute massive aortic insufficiency. Catheter Cardiovasc Interv.

[19] Cribier AG (2014). The Odyssey of TAVR from concept to clinical reality. Tex Heart Inst J.

[20] Gatto L, Biondi-Zoccai G, Romagnoli E, Frati G, Prati F, Giordano A (2018). New-generation devices for transcatheter aortic valve implantation. Minerva Cardioangiol.

