# Supplementary material for: Features and outcomes of bailout repeat transcatheter aortic valve implantation (TAVI): the Bailout Acute TAVI-in-TAVI to Lessen Events (BATTLE) international registry
Source: Clin Res Cardiol. 2023 Jun 9;113(1):68–74. doi: 10.1007/s00392-023-02239-8 (PMC10808138; doi:10.1007/s00392-023-02239-8)

**Features and outcomes of bailout repeat transcatheter aortic valve implantation (TAVI): evidence from the Bailout Acute TAVI-in-TAVI to Lessen Events (BATTLE) international registry**

**Online supplement**

**Table 1S.** Baseline imaging features. TAVI=transcatheter aortic valve implantation.

| **Features** | **Control** | **TAVI-in-TAVI** | **P value** |
| --- | --- | --- | --- |
| Patients | 212 | 106 | - |
| Aortic valve area (cm^2^) | 0.66±0.21 | 0.69±0.25 | 0.298 |
| Peak aortic valve gradient (mm Hg) | 79±25 | 78±77 | 0.782 |
| Mean aortic valve gradient (mm Hg) | 49±17 | 48±18 | 0.754 |
| Bicuspid aortic valve | 9 (4.3%) | 4 (3.8%) | 1 |
| Significant aortic valve calcification | 111 (52.4%) | 76 (71.7%) | 0.001 |
| Aortic regurgitation |  |  | 0.161 |
| None or trivial | 151 (71.2%) | 77 (72.6%) |  |
| Mild | 44 (20.8%) | 18 (17.0%) |  |
| Moderate | 16 (7.6%) | 7 (6.6%) |  |
| Moderate to severe | 1 (0.5%) | 4 (3.8%) |  |
| Maximum aortic anulus diameter (mm) | 26±4 | 26±4 | 0.840 |
| Sinus of Valsalva diameter (mm) | 31±4 | 33±4 | 0.014 |
| Sino-tubular junction diameter (mm) | 28±3 | 30±5 | 0.008 |
| Ascending aortic diameter (mm) | 34±5 | 36±5 | 0.014 |
| Porcelain aorta | 1 (0.5%) | 0 | 1 |
| Left ventricular end-diastolic diameter (mm) | 48±7 | 49±9 | 0.179 |
| Left ventricular end-systolic diameter (mm) | 34±9 | 34±9 | 0.812 |
| Septal thickness (mm) | 14±3 | 14±3 | 0.401 |
| Posterior wall thickness (mm) | 13±2 | 12±2 | 0.149 |
| Left ventricular ejection fraction (%) | 55±11 | 53±13 | 0.314 |
| Left ventricular outflow diameter (mm) | 20±3 | 20±3 | 0.351 |
| Low flow-low gradient aortic stenosis | 7 (3.3%) | 10 (9.4%) | 0.032 |
| Mitral regurgitation |  |  | 0.971 |
| None or trivial | 34 (16.0%) | 15 (14.2%) |  |
| Mild | 101 (47.6%) | 54 (50.9%) |  |
| Moderate | 69 (32.6%) | 34 (32.1%) |  |
| Moderate to severe | 4 (1.9%) | 1 (0.9%) |  |
| Severe | 4 (1.9%) | 2 (1.9%) |  |
| Systolic pulmonary artery pressure (mm Hg) | 43±13 | 42±11 | 0.514 |

**Table 2S.** Reasons for TAVI-in-TAVI. TAVI=transcatheter aortic valve implantation.

| **Features** | **Count** | **%** |
| --- | --- | --- |
| Cranial embolization | 10 | 9.4% |
| Low implantation | 3 | 2.8% |
| Significant aortic regurgitation or paravalvular leak | 9 | 8.5% |
| Not reported | 84 | 79.3% |

**Table 3S.** Devices used for TAVI-in-TAVI. TAVI=transcatheter aortic valve implantation.

|  | | **Second device** | | | | | | | |
| --- | --- | --- | --- | --- | --- | --- | --- | --- | --- |
|  |  | **Acurate** | **Allegra** | **CoreValve** | **Evolut** | **Jena Valve** | **Myval** | **Portico/ Navitor** | **Sapien** |
| **First device** | **Accurate** | 4 (30.8%) | 0 | 0 | 3 (23.1%) | 0 | 0 | 0 | 6 (46.2%) |
|  | **Allegra** | 0 | 3 (60.0%) | 0 | 2 (40.0%) |  | 0 | 0 | 0 |
|  | **CoreValve** | 0 | 0 | 36 (97.3%) | 1 (2.7%) | 0 | 0 | 0 | 0 |
|  | **Evolut** | 0 | 0 | 1 (3.5%) | 24 (82.8%) | 0 | 0 | 0 | 4 (13.8%) |
|  | **Jena Valve** | 0 | 0 | 0 | 0 | 1 (50.0%) | 0 | 0 | 1 (50.0%) |
|  | **Myval** | 0 | 0 | 0 | 0 | 0 | 2 (100%) | 0 | 0 |
|  | **Portico/ Navitor** | 0 | 0 | 0 | 0 | 0 | 1 (11.1%) | 7 (77.8%) | 1 (11.1%) |
|  | **Sapien** | 0 | 0 | 1 (11.1%) | 0 | 0 | 0 | 0 | 8 (88.9%) |

**Table 4S.** Postpocedural and inhospital outcomes. TAVI=transcatheter aortic valve implantation.

| **Features** | **Control** | **TAVI-in-TAVI** | **P value** |
| --- | --- | --- | --- |
| Patients | 212 | 106 | - |
| Postprocedural echocardiogram |  |  |  |
| Peak aortic valve gradient (mm Hg) | 17±11 | 14±8 | 0.064 |
| Mean aortic valve gradient (mm Hg) | 9±5 | 8±5 | 0.085 |
| Aortic regurgitation |  |  | 0.196 |
| None or trivial | 122 (60.4%) | 57 (55.3%) |  |
| Mild | 66 (32.7%) | 34 (33.0%) |  |
| Moderate | 1 (0.5%) | 4 (4.9%) |  |
| Moderate to severe | 1 (0.5%) | 0 |  |
| Mitral regurgitation |  |  | 0.464 |
| None or trivial | 87 (54.4%) | 37 (43.0%) |  |
| Mild | 50 (31.3%) | 32 (37.2%) |  |
| Moderate | 16 (10.0%) | 11 (12.8%) |  |
| Moderate to severe | 5 (3.1%) | 4 (4.7%) |  |
| Severe | 2 (1.3%) | 2 (2.3%) |  |
| Discharge echocardiogram |  |  |  |
| Peak aortic valve gradient (mm Hg) | 17±9 | 16±8 | 0.318 |
| Mean aortic valve gradient (mm Hg) | 9±5 | 9±5 | 0.305 |
| Aortic regurgitation |  |  | 0.112 |
| None or trivial | 116 (55.5%) | 51 (56.7%) |  |
| Mild | 79 (37.8%) | 29 (32.2%) |  |
| Moderate | 14 (6.7%) | 7 (7.8%) |  |
| Moderate to severe | 0 | 2 (2.2%) |  |
| Severe | 0 | 1 (1.1%) |  |
| Mitral regurgitation |  |  | 0.720 |
| None or trivial | 24 (14.1%) | 7 (8.8%) |  |
| Mild | 98 (57.7%) | 48 (60.0%) |  |
| Moderate | 37 (21.8%) | 19 (23.8%) |  |
| Moderate to severe | 7 (4.1%) | 5 (6.3%) |  |
| Severe | 4 (2.4%) | 1 (1.3%) |  |
| Systolic pulmonary artery pressure (mm Hg) | 40±13 | 40±11 | 0.803 |
| Hospital stay (days) | 6±6 | 7±6 | 0.153 |
| Inhospital outcomes |  |  |  |
| Death | 3 (1.4%) | 16 (15.1%) | <0.001 |
| Myocardial infarction | 1 (0.5%) | 1 (0.9%) | 1 |
| Stroke | 3 (1.4%) | 3 (2.8%) | 0.404 |
| Access site complication | 33 (15.6%) | 17 (16.0%) | 1 |
| Bleeding | 43 (20.3%) | 27 (25.5%) | 0.316 |
| Significant coronary obstruction | 3 (1.4%) | 0 | 0.553 |
| Major bleeding | 23 (10.9%) | 15 (14.2%) | 0.463 |
| Emergency surgery | 1 (0.5%) | 4 (3.8%) | 0.044 |
| Pacemaker implantation | 20 (9.4%) | 23 (21.7%) | 0.005 |
| Tamponade | 3 (1.4%) | 5 (4.7%) | 0.123 |
| Major adverse event* | 48 (22.6%) | 41 (38.7%) | 0.003 |

*composite of death, myocardial infarction, stroke, major bleeding, or reintervention

**Table 5S.** Follow-up assessments. TAVI=transcatheter aortic valve implantation.

| **Features** | **Control** | **TAVI-in-TAVI** | **P value** |
| --- | --- | --- | --- |
| 1-month follow-up |  |  |  |
| Patients | 110 | 64 | - |
| New York Heart Association |  |  | 0.643 |
| I | 58 (52.7%) | 32 (50.0%) |  |
| II | 40 (36.4%) | 28 (43.8%) |  |
| III | 10 (9.1%) | 4 (6.3%) |  |
| IV | 2 (1.8%) | 0 |  |
| Peak aortic valve gradient (mm Hg) | 18±10 | 16±9 | 0.326 |
| Mean aortic valve gradient (mm Hg) | 10±5 | 9±5 | 0.194 |
| Aortic regurgitation |  |  | 0.829 |
| None or trivial | 43 (49.4%) | 29 (53.7%) |  |
| Mild | 34 (39.1%) | 18 (33.3%) |  |
| Moderate | 10 (11.5%) | 7 (13.0%) |  |
| Moderate to severe | 0 | 0 |  |
| Mitral regurgitation |  |  | 0.638 |
| None or trivial | 9 (12.2%) | 8 (15.7%) |  |
| Mild | 43 (58.1%) | 27 (52.9%) |  |
| Moderate | 14 (18.9%) | 11 (21.6%) |  |
| Moderate to severe |  |  |  |
| Severe |  |  |  |
| Systolic pulmonary artery pressure (mm Hg) | 40±16 | 39±17 | 0.635 |
| 12-month follow-up |  |  |  |
| Patients | 127 | 61 | - |
| New York Heart Association |  |  | 0.774 |
| I | 55 (43.3%) | 28 (45.9%) |  |
| II | 54 (42.5%) | 28 (45.9%) |  |
| III | 14 (11.0%) | 4 (6.6%) |  |
| IV | 4 (3.2%) | 1 (1.6%) |  |
| Peak aortic valve gradient (mm Hg) | 19±9 | 18±9 | 0.615 |
| Mean aortic valve gradient (mm Hg) | 10±6 | 10±5 | 0.436 |
| Aortic regurgitation |  |  | 0.790 |
| None or trivial | 31 (40.3%) | 19 (37.3%) |  |
| Mild | 36 (46.8%) | 27 (52.9%) |  |
| Moderate | 10 (13.0%) | 5 (9.8%) |  |
| Moderate to severe | 0 | 0 |  |
| Mitral regurgitation |  |  | 0.722 |
| None or trivial | 9 (11.4%) | 8 (16.0%) |  |
| Mild | 48 (60.8%) | 28 (56.0%) |  |
| Moderate | 19 (24.1%) | 11 (22.0%) |  |
| Moderate to severe | 2 (2.5%) | 3 (6.0%) |  |
| Severe | 1 (1.3%) | 0 |  |
| Systolic pulmonary artery pressure (mm Hg) | 40±12 | 39±13 | 0.722 |

**Table 6S.** Long-term outcomes censoring events occurring <1 month. TAVI=transcatheter aortic valve implantation.

| **Features** | **Control** | **TAVI-in-TAVI** | **P value** |
| --- | --- | --- | --- |
| Patients | 137 | 62 | - |
| Follow-up (months) | 2.8±2.4 | 3.0±2.5 | 0.583 |
| Death | 68 (37.8%) | 35 (44.9%) | 0.333 |
| Cardiovascular death | 41 (22.8%) | 15 (19.2%) | 0.623 |
| Myocardial infarction | 5 (4.0%) | 1 (2.1%) | 1 |
| Stroke | 10 (7.9%) | 2 (4.3%) | 0.517 |
| Bleeding | 0 | 1 (2.1%) | 0.272 |
| Major bleeding | 0 | 0 | - |
| Aortic valve reintervention | 1 (0.8%) | 0 | 1 |
| Major adverse event* | 49 (38.9%) | 19 (40.4%) | 0.863 |

*composite of death, myocardial infarction, stroke, major bleeding, or aortic valve reintervention

**Table 7S.** Unadjusted, adjusted and inverse probability of treatment weighted analyses for death and major adverse event. HR=hazard ratio; OR=odds ratio; TAVI=transcatheter aortic valve implantation.

| **Features** | **Unadjusted analysis** | **Adjusted analysis*** | **Inverse probability of treatment weighted analysis** |
| --- | --- | --- | --- |
| Inhospital |  |  |  |
| Death | OR=12.39 (3.52-43.56), p<0.001 | OR=16.24 (2.01-131.44), p=0.009 | OR=10.45 (4.19-26.03), p<0.001 |
| Major adverse event | OR=2.16 (1.30-3.58), p=0.003 | OR=2.34 (1.26-4.36), p=0.007 | OR=2.10 (1.44-3.05), p<0.001 |
| Cumulative at long-term |  |  |  |
| Death | HR=1.55 (1.09-2.20), p=0.015 | HR=1.93 (1.19-3.12), p=0.007 | HR=1.37 (1.04- 1.81), p=0.025 |
| Major adverse event | HR=1.46 (1.07- 1.99), p=0.018 | HR=1.80 (1.19- 2.72), p=0.005 | HR=1.38 (1.10- 1.74), p=0.005 |
| At long-term after censoring 1-month events |  |  |  |
| Death | HR=1.15 (0.76-1.72), p=0.513 | HR=1.68 (0.96- 2.93), p=0.068 | HR=1.02 (0.75-1.39), p=0.897 |
| Major adverse event | HR=1.18 (0.69-2.01), p=0.548 | HR=1.97 (0.75-5.19), p=0.171 | HR=1.11 (0.71-1.73), p=0.645 |

*adjusting for age, gender, body mass index, diagnosis, logistic EuroSCORE, EuroSCORE II, baseline New York Heart Association, approach, use of a balloon-expandable valve, and postdilation; OR or HR <1 favor TAVI-in-TAVI, OR or HR >1 favor control.

**Figure 1S.** Cumulative failure curves for death (top) and major adverse event (bottom). TAVI=transcatheter aortic valve implantation.


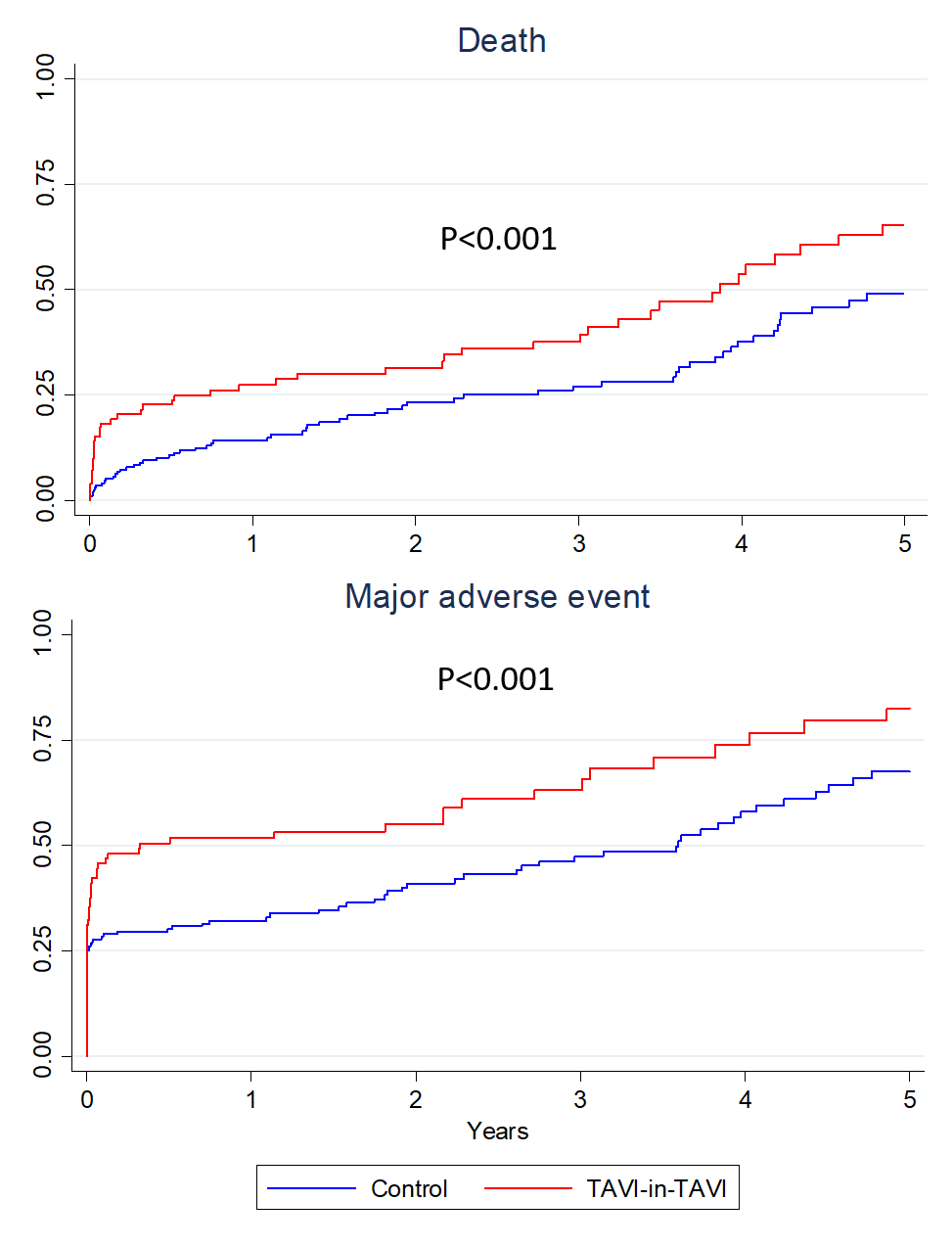

Supplement: Supplementary file 1 — Supplementary file1 (DOCX 92 kb) [file 392_2023_2239_MOESM1_ESM.docx]
